# Case of rheumatic mitral stenosis with bilateral coronary artery fistula to pulmonary artery: A rare entity

**DOI:** 10.34172/jcvtr.2021.13

**Published:** 2021-01-30

**Authors:** Manish Jawarkar, Pratik Manek, Mausam Shah, Vivek Wadhawa, Chirag Doshi, Divyesh Rathod

**Affiliations:** Department of Cardiovascular and Thoracic Surgery, Gujarat, India

**Keywords:** Coronary Arteriovenous Fistula, Mitral Stenosis

## Abstract

Coronary to pulmonary artery fistula is a rare form of congenital coronary artery anomaly. Majority of coronary arteriovenous fistula detected incidentally on coronary angiography. Although, most of these patients are asymptomatic, larger fistulae can produce symptoms of heart failure. Here we present a rare case of 61-year-old female who presented primarily for mitral valve replacement for severe mitral stenosis. On screening angiography, there were two fistula arising from both right and left coronary artery and draining in to the main pulmonary artery. The patient was operated and mitral valve replacement with closure of the fistula. Patient had an uneventful post-operative period and was discharged on 7 the post-operative day.

## Introduction

Coronary to pulmonary artery fistula is a rare form of congenital coronary artery anomaly. Majority of coronary arteriovenous fistula detected incidentally on coronary angiography. Although, most of these patients are asymptomatic, larger fistulae can produce symptoms of heart failure. Here we present a rare case of 61-year-old female who presented primarily for mitral valve replacement for severe mitral stenosis. On screening angiography, there were two fistula arising from both right and left Coronary artery and draining in to the main pulmonary artery. The patient was operated and mitral valve replacement with closure of the fistula. Patient had an uneventful post-operative period and was discharged on 7 the post-operative day.

## Case Presentation

**Figure 1:**
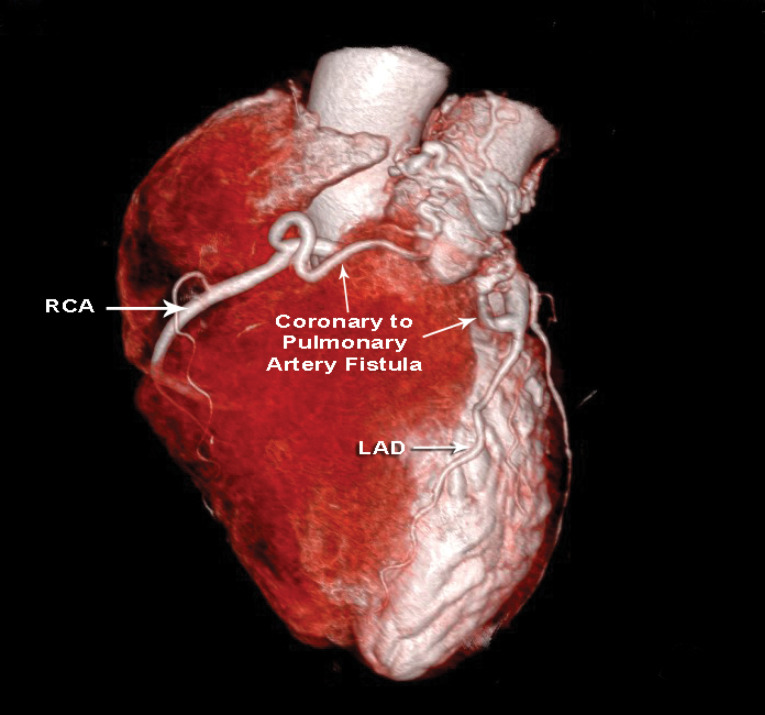
Mesh of the vessels over the pulmonary artery

A 61-year-old female presented to us with complaints of easy fatigability and dyspnea on exertion (New York Heart Association- (NYHA) Class 2) since last 4 months. The symptoms had gradually progressed over time. On examination she had an irregularly irregular pulse rate of around 96/min with a blood pressure of 106/72 mm hg. On auscultation a mid diastolic murmur was audible over the left 4th intercostal space. Transthoracic echocardiography confirmed the diagnosis of Severe Mitral stenosis with mild mitral regurgitation of rheumatic etiology. There was severe sub valvular disease with mitral valve area of 0.7 cm2. Pulmonary artery pressure was 48/26 mm Hg suggestive of mild pulmonary arterial hypertension (PAH). Ejection fraction was 55% and left ventricular (LV) dimensions were as follows: left ventricular end diastolic diameter (LVEDD)/left ventricular end systolic diameter (LVESD) = 47/31 mm. So we decided to do a mitral valve replacement. A written informed consent was taken from the patient. A screening Coronary Angiography was performed which showed an incidental finding of two coronary artery fistulae, one from left anterior descending artery (LAD) draining to pulmonary artery and one from RCA possibly draining in to the right atrium. A CT coronary angiography was performed to further properly delineate the anatomy of the fistulae. The CT angiography revealed fistulae arising from both proximal right coronary artery (RCA) and proximal LAD with contributions of few collaterals arising from aorta directly to form a plexus which drained from a single opening on the left lateral aspect of main pulmonary artery (MPA) (Figure 1).

**Figure 2:**
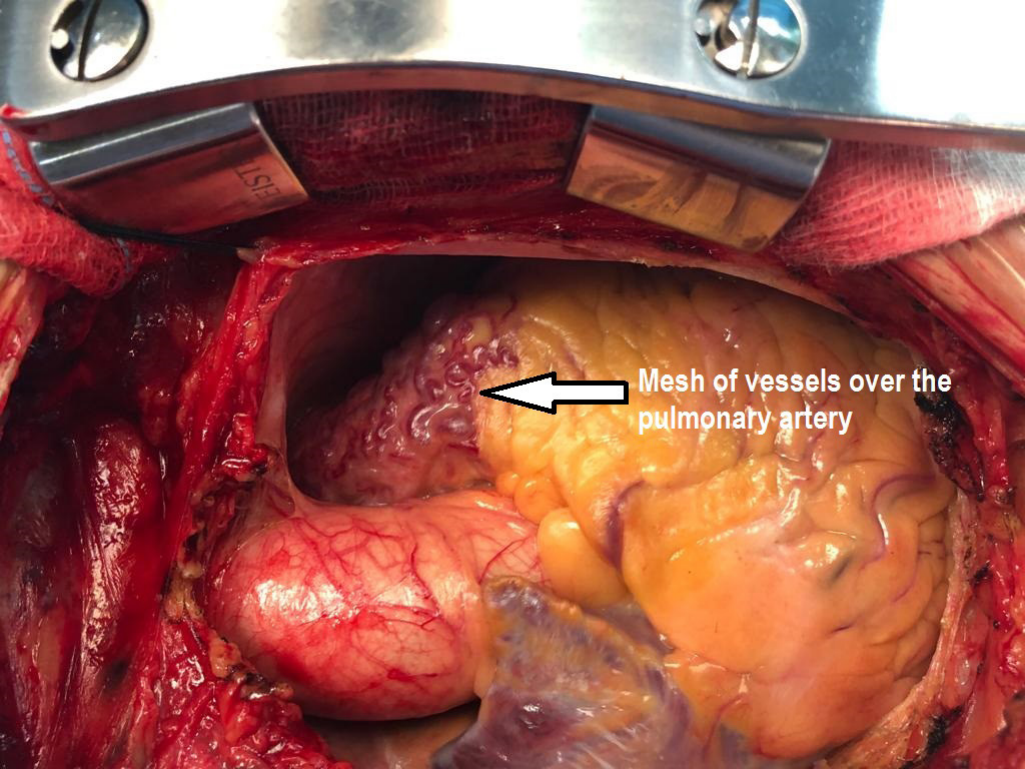
Internal opening of the fistula

**Figure 3:**
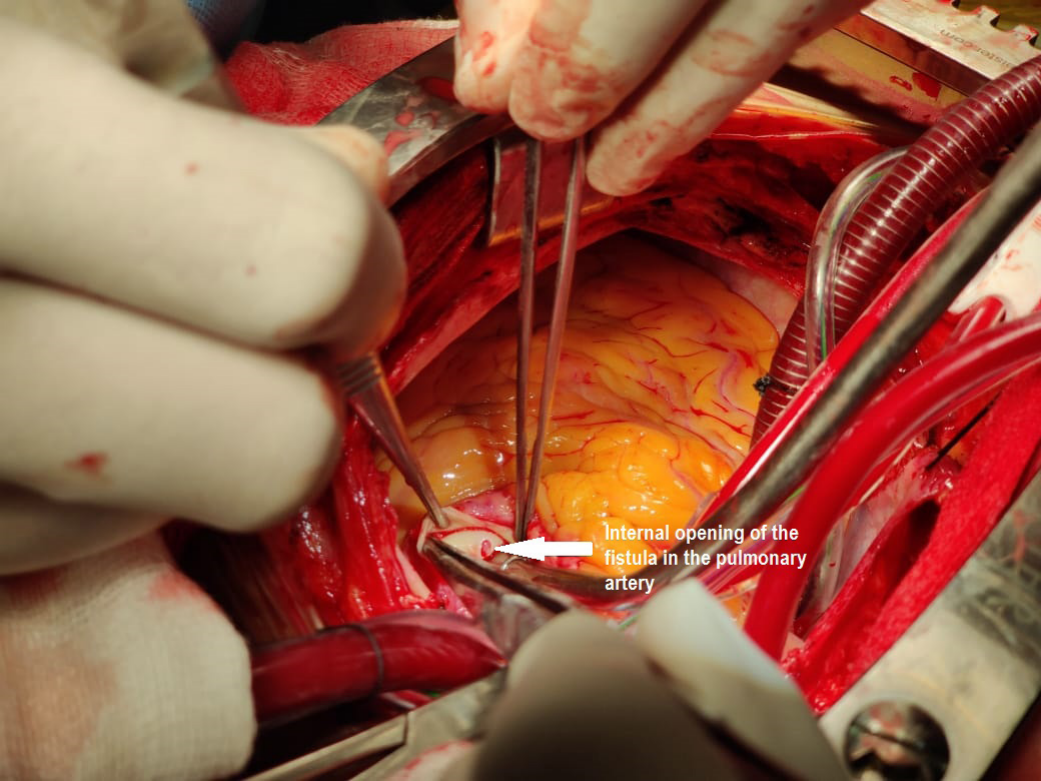
Internal opening of the fistula in the pulmonary artery

**Figure 4:**
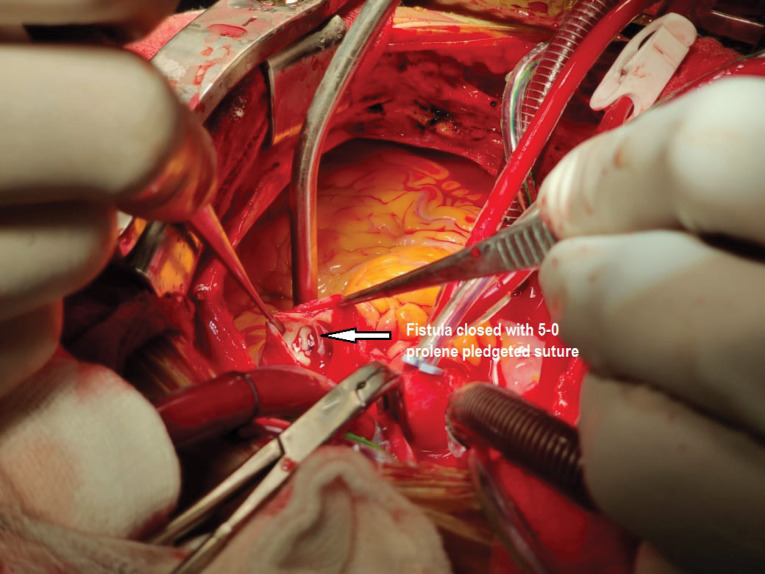
Fistula closed with 5-0 prolene pledgeted suture

On opening up the chest there were multiple collaterals seen over the anterior surface of pulmonary (Figure 2). Patient was put on cardiopulmonary bypass using aortic and bicaval cannulation. Cross clamp was applied on the aorta to include the pulmonary artery as well to prevent the cardioplegia to run off. Using the CT Scan findings which showed the internal opening of the fistula to be on the left lateral aspect of the MPA. A 4 cm longitudinal incision was placed on the pulmonary artery. A 2 mm opening was seen on the left lateral aspect of the pulmonary artery (Figure 3). No other opening was seen. On administering cardioplegia, it was seen to drain through the opening. The opening was closed with 5-0 prolene pledegeted suture (Figure 4). The closure of fistula was tested by running the cardioplegia solution. No leakage was seen in the PA. Pulmonary artery was closed in double layer using double layer 6-0 prolene sutures. Mitral valve replacement was done using 27 mm St jude Masters mechanical valve in usual manner. Patient was weaned off bypass and shifted to recovery with good hemodynmics. Patient had an uneventful postoperative period and was discharged on the 7th post-operative day.

## Discussion

The Coronary artery fistula is relatively rare and is seen in only 0.1%-0.2% of coronary angiograms^1^. The right coronary artery or its branches are the site of the fistula in around 50% of cases. Left coronary artery is involved in around 35%, and both the coronary arteries in around 5% cases. Most common site of termination of the fistula are right ventricle in 40%, right atrium in 25%, to pulmonary artery in 15%-20%, 7% to coronary sinus and around 1% to superior vena cava. Our patient had two fistulae, likely congenital, one originating from LAD and one arising from RCA, with both of them draining via a common channel in to the pulmonary artery, which is less commonly reported^2,3^. Coronary artery anomalies can be divided in to 3 types: abnormalities of origin, distribution and termination. Of these, coronary artery fistula is believed to be termination abnormalities^4^.

Most of the patients with isolated coronary artery fistula are asymptomatic and are detected incidentally. Large fistula can present with heart failure, dypnoea and angina. Infective endocarditis can also precipitate symptoms. Other complications include formation of aneurysm and spontaneous rupture. Our patient did not have any symptoms as far as the fistula is considered. But we decided to address the fistula as the patient had been put on cardio-pulmonary bypass for mitral valve replacement. Further, it was felt that if we did not address the fistula at operation then there was a chance that following mitral valve replacement, with the reduction in the pulmonary artery pressures the shunting from the fistula may increase and leads to volume overload of the left ventricle^5^. Diagnosis of coronary arteriovenous fistula is itself an indication of surgical intervention as most of these fistulae will increase in size causing symptoms of heart failure or lead to development of infective endocarditis. Also, the probability of spontaneous closure is less.

## Conclusion

Although the surgical procedure was essentially the same as used for routine mitral valve replacement, care was taken to cross-clamp the pulmonary artery along with the aorta to prevent the cardioplegia running off in to the pulmonary artery. Also, as there was a leash of vessels overlying the pulmonary artery, decision was taken to ligate the fistula internally by opening the pulmonary artery.

## Competing interests

The authors declare no conflict of interest.

## Ethical approval

Informed and written consent was taken from the patient for the present case report. This was provided on the basis that patient’s name and other details will remain confidential.
